# Nicotine but not saline self-administering or yoked control conditions produces sustained neuroadaptations in the accumbens shell

**DOI:** 10.3389/fnmol.2023.1105388

**Published:** 2023-01-25

**Authors:** Ana Domi, Erika Lucente, Davide Cadeddu, Louise Adermark

**Affiliations:** ^1^Department of Pharmacology, Institute of Neuroscience and Physiology, The Sahlgrenska Academy, University of Gothenburg, Gothenburg, Sweden; ^2^Addiction Biology Unit, Department of Psychiatry and Neurochemistry, Institute of Neuroscience and Physiology, The Sahlgrenska Academy, University of Gothenburg, Gothenburg, Sweden

**Keywords:** nicotine, electrophysiology, whole-cell recording, operant self-administration, abstinence, conditioned stimuli (cues), smoking

## Abstract

**Introduction:**

Using yoked animals as the control when monitoring operant drug-self-administration is considered the golden standard. However, instrumental learning *per se* recruits several neurocircuits that may produce distinct or overlapping neuroadaptations with drugs of abuse. The aim of this project was to assess if contingent responding for nicotine or saline in the presence of a light stimulus as a conditioned reinforcer is associated with sustained neurophysiological adaptations in the nucleus accumbens shell (nAcS), a brain region repeatedly associated with reward related behaviors.

**Methods:**

To this end, nicotine-or saline-administrating rats and yoked-saline stimulus-unpaired training conditions were assessed in operant boxes over four consecutive weeks. After four additional weeks of home cage forced abstinence and subsequent cue reinforced responding under extinction conditions, *ex vivo* electrophysiology was performed in the nAcS medium spiny neurons (MSNs).

**Results:**

Whole cell recordings conducted in voltage and current-clamp mode showed that excitatory synapses in the nAcS were altered after prolonged forced abstinence from nicotine self-administration. We observed an increase in sEPSC amplitude in animals with a history of contingent nicotine SA potentially indicating higher excitability of accumbal MSNs, which was further supported by current clamp recordings. Interestingly no sustained neuroadaptations were elicited in saline exposed rats from nicotine associated visual cues compared to the yoked controls.

**Conclusion:**

The data presented here indicate that nicotine self-administration produces sustained neuroadaptations in the nAcS while operant responding driven by nicotine visual stimuli has no long-term effects on MSNs in nAcS.

## Introduction

Self-administration (SA) of drugs of abuse has been widely used as a preclinical investigative tool to study behavioral, neurobiological, and genetic factors associated with drug addiction (Spealman and Goldberg, 1978; Panlilio and Goldberg, 2007). Besides the drug consummatory response, operant self-administration paradigms evaluate, appetitive and motivational components of drug seeking, taking and relapse (for review see Belin-Rauscent et al., 2016). Nicotine, the main component of tobacco smoke, is highly addictive in humans and intravenous nicotine self-administration (IVSA) is considered the gold standard for studying the rewarding and motivational aspects of the drug in rodents (Donny et al., 1995; Palmatier et al., 2006; Henningfield et al., 2016).

A widely-used procedure for demonstrating specificity of effect during drug self-administration sessions is to include a yoked control condition that passively receives either the psychoactive drug or saline (Lecca et al., 2007; Mathieson et al., 2022). Generally, a yoked-saline design is used as a control when assessing for the pharmacological effects of nicotine and possible neuroadaptations elicited by the drug (Palmatier et al., 2007; Gipson et al., 2013b; Pittenger et al., 2018; Jin et al., 2020). However, more than in any other drug of abuse, the reinforcing properties of nicotine are strongly driven by paired sensory stimuli that through associative learning processes maintain smoking behavior (Caggiula et al., 2001; Bevins and Palmatier, 2004; Markou and Paterson, 2009). In the yoked-saline control there is not Pavlovian conditioning and instrumental performance such as nose-poking or pressing a lever has no consequences. Conversely, a saline self-administering group can be used to control for neurophysiological transformations that may be shielded by neuroadaptations produced when conditioned visual cues are associated with operant responding. As a matter of fact, saline self-administering rats that respond for nicotine associated visual cues, present a stimulus-driven operant learning and are able to discriminate between active (rewarded) and inactive (non-rewarded) responses (Donny et al., 2003; Garcia-Rivas et al., 2019).

When learning to lever press for nicotine in the presence of contingent visual stimuli, several brain regions involved in instrumental learning or stimulus–response associations are recruited (for review see Schmidt et al., 2019). In particular the nucleus accumbens (nAc) appears to play a central role in the way reward-related cues influence instrumental performance (Corbit et al., 2001; Di Ciano et al., 2008). Lesions and pharmacological manipulations of the nAc impair the ability of conditioned stimuli (CS) to promote operant responding for psychostimulants (Ito et al., 2004; Di Ciano et al., 2008; Floresco et al., 2008). For instance, response-contingent nicotine SA paired with a visual stimulus preferentially increases the dopamine output in the nAc shell (nAcS) as compared to that in the core subdivision, at different training periods but not following extinction (Lecca et al., 2006).

Changes in dopaminergic activity in the nAcS seem to involve mGluR 2/3 receptors signaling (Karasawa et al., 2006; Liechti et al., 2007; Karasawa et al., 2010) and during nicotine intake and withdrawal there is an interplay between compensatory mechanisms involving mGluR 2/3 and AMPA/kainite receptor within the mesolimbic dopamine pathway (Kenny et al., 2003; Liechti et al., 2007). In fact, mGluR2/3 in nAcS has been proposed to be involved in mediating the rewarding effects of nicotine and potentially also in cue-induced nicotine-seeking behavior (Liechti et al., 2007). Furthermore, accumbal AMPA receptor signaling has been shown to play a role both cue-induced nicotine seeking behavior (Gipson et al., 2013b), as well as cocaine seeking behavior (Gipson et al., 2013a), and changes in calcium-permeable AMPARs are associated with incubation of cocaine and methamphetamine craving (Conrad et al., 2008; Scheyer et al., 2016; Murray et al., 2019). Studies outlining nicotine-induced glutamatergic neuroplasticity in the accumbens may thus be important for defining neurobiological mechanisms underlying reinstatement to nicotine-cues.

In the present study we assessed long-lasting changes in nAcS neurotransmission elicited by nicotine operant self-administration in the presence of environmental stimuli. We controlled both for the pharmacological effects of nicotine using yoked-saline rats and for the effects of the nicotine associated visual cues using a group that self-administered saline in an identical environment. *Ex vivo* whole-cell patch-clamp and field potential electrophysiological recordings were conducted in the nAcS 1 month after discontinuing nicotine self-administration.

## Materials and methods

### Animals

Male Wistar rats (Charles River, Germany), 150–170 g at arrival, were paired-housed in a temperature and humidity-controlled environment under a reversed 12:12-h light/dark cycle (lights off at 8:00 a.m.) with food and water *ad libitum*. Rats were habituated to the facility and handled prior to experiments. Experiments were conducted during the dark phase of the cycle and all efforts were made to minimize rats’ suffering and distress. Procedures were conducted in accordance with the National Committee for Animal Research in Sweden and approved by the Local Ethics Committee for Animal Care and Use at Gothenburg University.

### Drugs

The (−)-Nicotine hydrogen tartrate salt (Sigma-Aldrich, St. Louis, MO) was dissolved in sterile physiological saline and administered intravenously at a concentration of 30.0 μg/kg/0.1 mL infusion. The pH of the solution was adjusted to 7.4 with NaOH 5 M. The GABAA receptor antagonist bicuculline-methiodide (bicuculline) was diluted in Milli-Q to 20 mM and further diluted in aCSF (20 μM). The NMDA receptor antagonist D-(−)-2-Amino-5-phosphonopentanoic acid (APV; 50 μM) and the AMPA receptor antagonist CNQX (10 μM) were dissolved in aCSF shortly before use. All drugs were purchased from Sigma Aldrich (Stockholm, Sweden).

### Catheter implantation

Chronic jugular intravenous catheter implantation was conducted as previously described (Domi et al., 2022). Briefly, animals were anesthetized with isoflurane anesthesia: 5% induction and 2% maintenance. For intravenous surgery, incisions were made to expose the right jugular vein and a catheter made from micro-renathane tubing (ID = 0.020′′, OD = 0.037″; Braintree Scientific) was positioned subcutaneously between the vein and the back. Rats were treated subcutaneously with 10 mg/kg of enrofloxacin (50 mg/mL, Baytril, Germany) for 3 days post-surgery and allowed 3 week of recovering before self-administration training. Catheters were daily flushed with 0.1–0.2 mL of sterile saline mixed with heparin (20 U/mL, Italfarmaco S.p.A, Milan, Italy) for the entire duration of the experiments. Patency of the catheters was confirmed by intravenous injection of 150 μL/rat of Sodium Pentothal (25 mg/mL, Intervet, Italy) at the end of experimental procedures.

### Operant training

#### SA apparatus

The self-administration stations consisted of operant conditioning chambers (29.5 cm × 32.5 cm × 23.5 cm; Med Associates, St. Albans, VT) enclosed in sound-attenuating, ventilated environmental cubicles. Each chamber was equipped with two retractable levers located in the front panel with two stimulus light placed above each lever, a house light at the top of the opposite panel and a tone generator. Infusions were delivered through a system composed by an infusion pump, swivel, counterbalanced arm assembly, tether and a plastic tube that was connected to the catheter before the beginning of the session. Activation of the pump by responses in the “active lever” resulted in a delivery of 0.1 mL of fluid while responses on the “inactive” lever were recorded but did not result in any programmed consequences. An IBM compatible computer controlled the delivery of fluids and the recording of the behavioral data with MED-PC® IV windows-compatible software.

#### Operant self-administration

A week after surgery rats (≈70 days old) were randomly assigned to self-administer either nicotine (30 μg/kg/0.1 mL infusion) or saline (0.9% NaCl) and a third yoked-saline group was yoked to the rats that self-administered nicotine. Rats in the nicotine-SA and saline-SA group were trained for 2 h/daily (5 days/week) under Fixed Ratio 1 (FR1) contingency in which every lever response resulted in the delivery of a single dose of nicotine or saline. After 7 days in FR1 the response requirement for each infusion was then incremented to FR3 to ensure stable nicotine self-administration rates for the remainder of the training. Each infusion was followed by the activation of a cue-light above the active lever for 5-s and a total of 20-s time out period (TO) where responses at the active lever were not reinforced. The stimulus light presented to the nicotine group (nicotine associated visual stimuli) was the same for the saline-SA and yoked-saline rats. The yoked-saline rats received the identical infusion and stimulus light onsets/offsets as the animals that learned to self-inject nicotine.

The entire duration of the 120-min session was signaled by the intermittent cue-tone (1 s ON/1 s OFF; 7 kHz, 70 dB) as previously described (Cippitelli et al., 2015).

#### Forced abstinence and cue-reinforced responding under extinction conditions

Following training rats entered the abstinence phase in which they were left undisturbed in their home cages for 28 days. In this forced abstinence period rats were handled daily and received standard care. After this period rats were subjected to a single session of cue-reinforced responding under extinction conditions for the duration of 2 h. Pressing on the active lever resulted in illumination of the cue light above the lever under an FR3 schedule but nicotine or saline were no longer delivered. Cue responding was measured as the number of responses on the active lever throughout the test session, including during the timeout periods. Inactive lever presses were also recorded as a measure of non-specific responding.

### Brain slice preparation

To obtain brain slices, rats were deeply anesthetized with isoflurane and decapitated. Brains were rapidly removed and transferred into a constantly oxygenated (95% O2, 5% CO2) modified artificial cerebrospinal fluid solution (aCSF) containing (in mM): 220 sucrose, 2 KCl, 0.2 CaCl2, 6 MgCl2, 26 NaHCO3, 1.3 NaH2PO4 and 10 D-glucose. Coronal brain slices (250 μm) containing the nAcS were obtained using a Leica VT 1200S Vibratome (Leica Microsystems AB, Bromma, Sweden), and submerged in a continuously oxygenated standard aCSF containing (in mM): 124 NaCl, 4.5 KCl, 2 CaCl2, 1 MgCl2, 26 NaHCO3, 1.2 NaH2PO4 and 10 D-glucose. After an incubation for 30 min in 33°C, slices were allowed to rest for additionally 30 min before electrophysiological recordings were performed. Slices were maintained at room temperature for the rest of the day.

### Field potential recordings

Glutamatergic plasticity has been linked to behavioral transformations elicited by prolonged withdrawal from psychostimulants (Conrad et al., 2008; Ping et al., 2008; Mameli et al., 2009; McCutcheon et al., 2011), and to outline if sustained neurophysiological transformations also would be present in the nAcS after a history of nicotine SA, electrophysiological field potential recordings were performed as previously described (Adermark et al., 2011; Licheri et al., 2019). In brief, population spikes (PS) were evoked with a stimulation frequency of 0.05 Hz in the nAc shell. Stimulation electrodes (type TM33B, World Precision Instruments, Sarasota, FL) were positioned locally, 0.2–0.3 mm from the recording electrode (borosilicate glass, 2.5 to 4.5 MΩ, World Precision Instruments), and the amplitude of PSs were measured. To assess long-lasting effects by treatment on synaptic output, stimulus response curves were conducted by stepwise increasing the afferent stimulation strength creating a stimulus/response curve. To estimate changes in the probability of transmitter release, responses were evoked with a paired pulse stimulation protocol (50 ms interpulse interval), and the paired pulse ratio (PPR) was calculated by dividing the second pulse (PS2) with the first pulse (PS1).

To monitor changes in inhibitory tone, slices were treated with bicuculline (20 μM). For these measurements, the stimulus intensity was set to yield a PS amplitude of approximately half the size of the maximal amplitude of the evoked response, and a stable baseline was monitored for 10 min before drug-perfusion. Following 20 min of bath perfusion, stimulus/response curves were recorded to assess changes in synaptic output.

### Whole-cell recordings

A Nikon Eclipse FN-1 microscope equipped with a 10x/0.30 objective identified the nAcS, and a 40×/0.80 water-immersion objective was used to identify medium spiny neurons (MSNs) for whole-cell recordings. Recording pipettes were prepared from borosilicate glass using a micropipette puller (Sutter Instruments, Novato, CA) with a resistance ranging from 2.5 to 5.5 MΩ. Pipettes were filled with an internal solution containing (in mM): 135 K-Glu, 20 KCl, 2 MgCl_2_, 0.1 EGTA, 10 Hepes, 2 Mg-ATP and 0.3 Na-GTP, pH adjusted to 7.3 with KOH, and osmolarity to 295 mOsm with sucrose. Whole-cell recordings were conducted under constant flow (2 mL/min) of standard aCSF at the temperature of 33°C–34°C. To record spontaneous excitatory postsynaptic currents (sEPSCs) in voltage clamp mode, neurons were clamped at-65 mV using a MultiClamp 700B amplifier (Molecular Devices, Axon CNS, San Jose, CA), digitized at 10 kHz and filtered at 2 kHz using Clampex (Molecular devices).

In a subpopulation of these neurons, after the voltage clamp recording, a current clamp protocol was applied. Current was injected with a duration of 1,000 ms and an increasing intensity (in intervals of 20 pA) from −80 to 160 pA in order to hyperpolarize and depolarize the neuronal membrane.

### Statistics and data analysis

We examined for significant violations for assumptions of homogeneity of variance by using Levene’s test. Behavioral and electrophysiological data were analyzed by analysis of variance (ANOVA) with factors for the respective analysis indicated in conjunction with its results. When appropriate, *post hoc* comparisons were performed using Newman–Keuls test. Paired or unpaired Student’s *t*-tests were used for statistical analysis when appropriate. Data were analyzed using STATISTICA, stat soft 13.0 (RRID:SCR_014213), Clampfit 10.2 (Molecular devices, Axon CNS, CA, United States), Minianalysis 6.0 (Synaptosoft), Microsoft Excel and GraphPad Prism 9 (GraphPad Software, San Diego, CA). All parameters are given as mean +/− SEM, and differences between groups were considered statistically significant at *p* < 0.05.

## Results

### Cue-maintained responding during training and reinstatement in nicotine and saline self-administering rats

**Figure 1:**
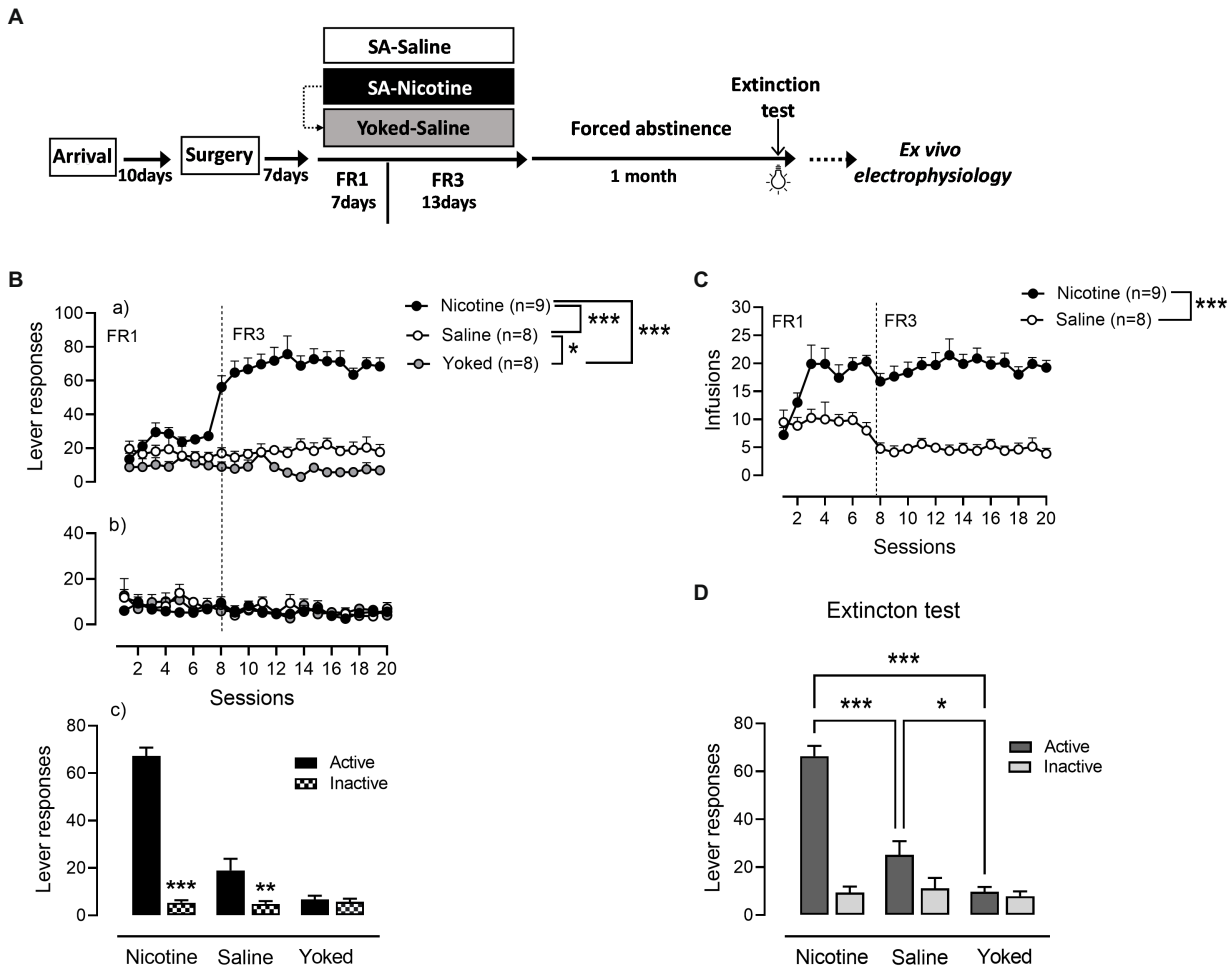
Cue-maintained responding during training and reinstatement in nicotine and saline self-administering rats. **(A)** Timeline of the experimental procedures that includes behavioral and neurophysiological assessments. **(B) (a)** During operant training nicotine-SA rats showed higher number of active lever presses over time compared to the control groups. Active responding for saline-SA rats was as well significantly higher than in yoked-saline control group. **(b)** Inactive lever pressing was very low with no differences between groups. **(c)** Both nicotine-SA and saline-SA rats discriminated between active and inactive lever presses while the yoked-saline group presented the same responding on both levers. **(C)** In accordance with the reported active lever presses, the number of infusions achieved over time was significantly higher in the rat self-administering nicotine compared to the saline-SA group. **(D)** Cue reinforced responding under extinction conditions 1 month after the last training session produced a significant higher operant responding in rats with a history of contingent nicotine-SA compared to saline-SA rats and yoked-saline control with the saline-SA group presenting higher active lever pressing than the yoked control, ^***^p < 0.001, ^**^p < 0.01, ^*^p < 0.01.

Nicotine (*n* = 9) and saline (*n* = 8) rats were trained to operant responding for 7 days under FR1 schedule of reinforcement followed by 13 days on FR3 (Figure 1). A third yoked-saline group (*n* = 8) underwent passive saline administration at the same time as the animals that learned to self-inject nicotine. Analysis of active lever responses over training showed a significant effect of group [Repeated measures ANOVA: main effect group *F*_(2,22)_ = 72.64, *p* < 0.0001, session *F*_(19,418)_ = 11.47, *p* < 0.0001 and group × session interaction *F*_(19,418)_ = 13.05, *p* < 0.0001]. As expected and demonstrated in Figures 1Ba, rats self-administering nicotine produced a higher number of active lever presses compared with the saline-SA and yoked-saline group (Newman–Keuls *post-hoc* test: N vs. S, *p* = 0.0014; N vs. Y, *p* = 0.0013). In addition, operant responding for saline produced significant increases in lever presses compared to the yoked-saline control group where lever presses were recorded but led to no consequences (S vs. Y, *p* = 0.0305). Inactive lever responses were very low and without differences between the three groups (main effect group: *F*_(2,22)_ = 0.3586, *p* = 0.7026; Figures 1Bb). However, while nicotine and saline rats were able to discriminate between levers [N_A_ (67.26 ± 3.51) vs. N_I_ (5.41 ± 1.02), *p* < 0.0001; S_A_ (19 ± 4.8) vs. S_I_ (4.83 ± 1.2), *p* = 0.0042] in yoked-saline rats the difference in pressing the active vs. the inactive lever was not significant [Y_A_ (6.75 ± 1.62) vs. Y_I_ (5.7 ± 1.34), *p* = 0.7860; Figures 1Bc]. This suggest that cue-contingency in saline-SA rats, present some intrinsic reinforcing properties in driving self-administration behavior. In accordance with the active lever responding data and the known pharmacological effects of nicotine, the number of self-infusions was significantly higher in the nicotine-SA than in the saline-SA rats [main effect group: *F*_(1,15)_ = 61.99, *p* < 0.0001; Figure 1C].

After a month of forced abstinence in their homecage rats were tested for cue-reinforced responding under extinction conditions (Figure 1D). An overall two-way ANOVA analysis yielded significant main effect of group: [*F*_(2,22)_ = 24.3, *p* < 0.0001], lever [*F*_(1,22)_ = 92.74, *p* < 0.0001] and a significant group × lever interaction [*F*_(2,22)_ = 45.57, *p* < 0.0001]. Rats with a history of nicotine SA exhibited a significant higher cue-reinforced responding as compared with rats trained with saline and yoked-saline group [N_A_ (66.33 ± 4.28) vs. S_A_ (25.13 ± 5.7), *p* = 0.0001; N_A_ (66.33 ± 4.28) vs. Y_A_ (9.75 ± 1.98), *p* = 0.0001]. In addition, cue-presentation alone during reinstatement produced a higher number of active lever presses in the saline-SA rats compared to yoked-saline rats that during training received yoked presentations of the same cue as the nicotine-SA group [S_A_ (25.13 ± 5.7) vs. Y_A_ (9.75 ± 1.98), *p* = 0.0495]. These results suggest that the conditioned-cue becomes motivationally salient not only if previously paired with a drug of abuse such as nicotine but as well with a neutral substance such as saline.

### Nicotine associated visual cue does not produce sustained neuroadaptations in the nAcS when paired with saline-SA rats

**Figure 2:**
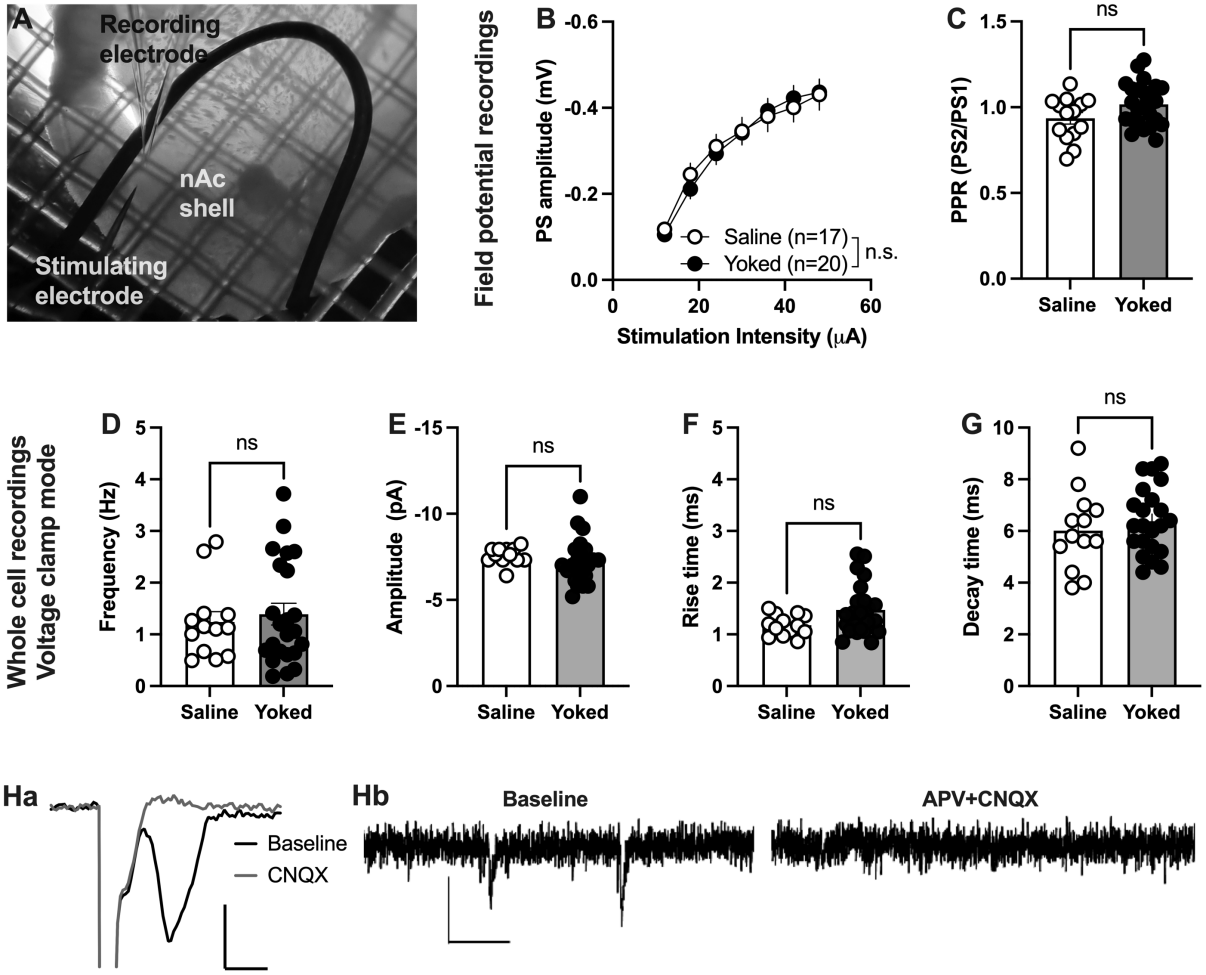
Neuronal transmission is not significantly altered in the nAcS of animals self-administering saline. **(A)** Micrograph showing the region of recording. **(B,C)** Evoked PS amplitudes were not significantly modulated by saline-self-administration, even though there was a trend toward increased PPR in brain slices from yoked-saline rats. **(D–G)** Whole cell recordings conducted in voltage clamp mode showed no effect by saline self-administration on neither frequency nor amplitude of spontaneous events. **(H)** Both evoked field potentials and sEPSCs were rapidly blocked by bath perfusion of CNQX and APV indicating that recorded activity is excitatory. Calibration: Ha: 2 ms, 0.2 mV. Hb: 50 ms, 10 pA. Values are presented as mean (±SEM), *n* = number of slices, taken from at least four animals.

During training and reinstatement rats responding for saline showed at a certain extent active lever pressing driven by nicotine visual stimuli as they distinguished between active and inactive lever. Thus, we first examined if operant responding maintained by environmental cues produces long-lasting neurophysiological transformations in the nAcS of saline-SA compared to the yoked-saline controls. To this end, electrophysiological recordings were conducted *ex vivo* in nAcS in brain slices from yoked-saline rats and saline-SA controls (Figure 2A) at least 2 days after the reinstatement test to avoid putative effects on transmission produced by the reinstatement session *per se*. Stimulus/response curves assessing evoked field potentials showed no differences between groups [main effect treatment: *F*_(1, 35)_ = 0.01195, *p* = 0.9136; time *F*_(6, 210)_ = 179.7, *p* < 0.001; time × drug: *F*_(6,210)_ = 1.258, *p* = 0.2783]. Even though there was a trend toward increased PPR in brain slices from yoked animals, the effect was not significant [Student’s *t*-test: *t*_(36)_ = 1.95, *p* = 0.060; Figures 2B, C]. CNQX blocked evoked potentials showing that recorded PS amplitudes are mediated through AMPA receptor activation (Figures 2Ha).

To further outline changes in accumbal neurotransmission induced by saline self-administration, whole cell recordings were conducted in medium spiny neurons. Recordings performed in voltage clamp mode demonstrated no effect by treatment on spontaneous activity. Neither the frequency [*t*_(33)_ = 0.3360, *p* = 0.7390], nor the amplitude [amplitude *t*_(33)_ = 1.342, *p* = 0.1888; decay time: *t*_(33)_ = 1.117, *p* = 0.2719; rise-time: *t*_(33)_ = 1.449, *p* = 0.1594] of spontaneous events were significantly affected (Figures 2D–G). CNQX and APV blocked all spontaneous events suggesting that all events are excitatory (Figures 2Hb).

### Nicotine self-administration followed by forced abstinence produces sustained neuroadaptations in nAc shell

Since there were no significant differences between yoked-saline and saline-SA control when assessing spontaneous activity in voltage-clamp mode, these groups were pooled to increase the number of animals assessed in each comparison. To examine if nicotine SA would produce more sustained neuroadaptations in the nAcS, we compared the control with nicotine-SA rats after 1 month of forced abstinence.

**Figure 3:**
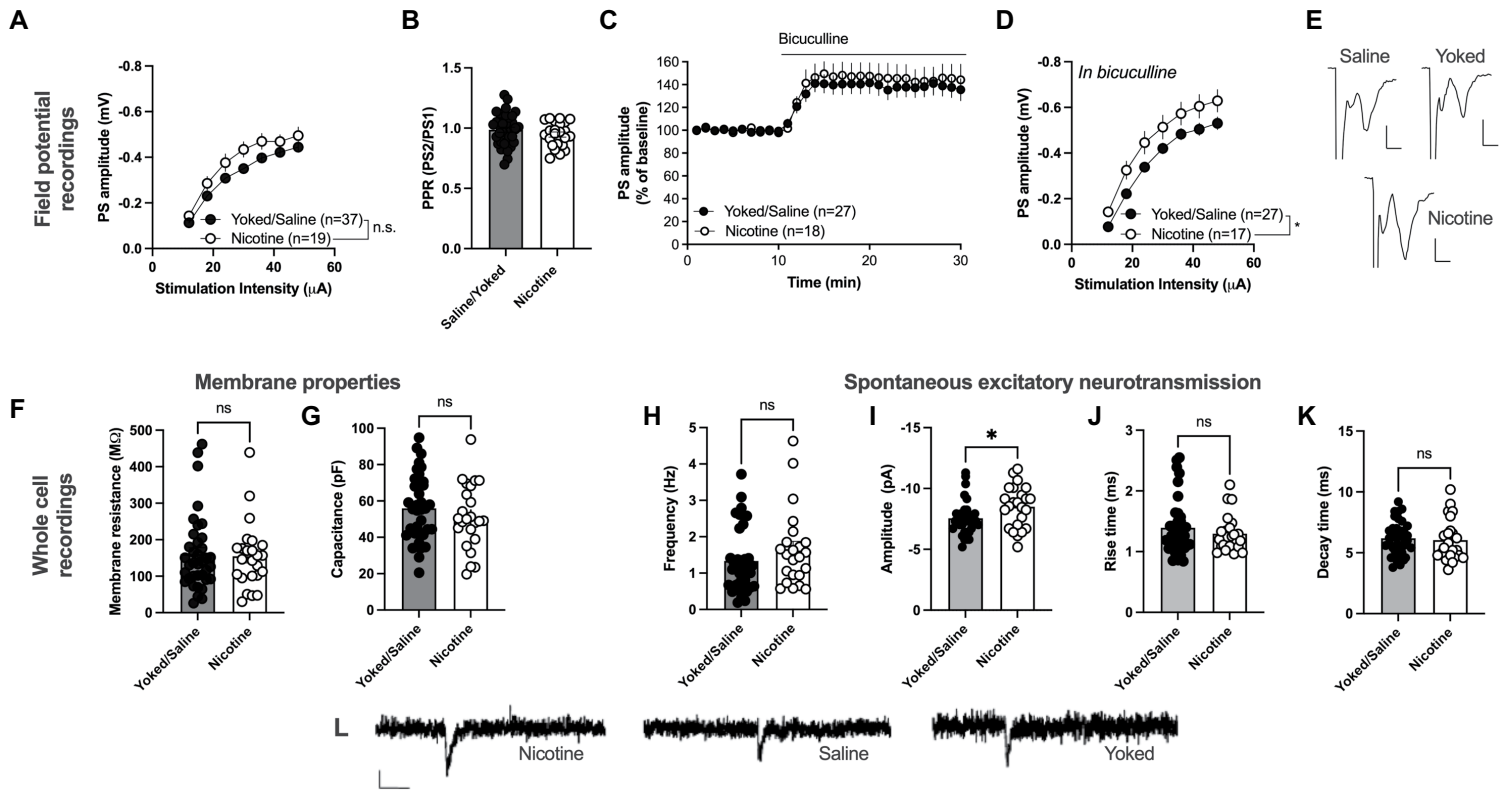
Nicotine SA increases excitatory neurotransmission in nAcS MSNs. **(A)** Field potential recordings demonstrated a trend toward potentiated stimulus–response curves in brain slices from nicotine-SA rats. **(B)** PPR was not modulated by nicotine-SA. **(C)** The GABAA receptor antagonist bicuculline disinhibited evoked potentials to a similar extent in both treatment groups. **(D)** Isolation of excitatory currents revealed a significantly enhanced stimulus–response curve in brain slices from nicotine-SA. **(E)** Example traces based on a mean of 5 traces at baseline for each treatment group. Calibration: 2 ms, 0.2 mV. **(F,G)** Membrane properties were not significantly modulated by treatment. **(H–K)** Recordings of spontaneous activity demonstrated a significant increase in sEPSC amplitude, indicating that nicotine-SA increases MSN responsiveness to excitatory inputs. **(L)** Example traces showing recorded sEPSCs in brain slices from all treatment groups. Calibration: 10 pA, 50 ms. Values are presented as mean (±SEM), *n* = number of slices, taken from at least four animals. ^*^p < 0.05.

Field potential recording revealed a trend toward an increase in PS amplitude in the nAc shell of rats self-administering nicotine [main effect treatment: *F*_(1, 54)_ = 3.199, *p* = 0.0793; time *F*_(6, 324)_ = 231.6, *p* < 0.001; time × drug *F*_(6,324)_ = 1.212, *p* = 0.2997; Figures 3A, E]. PPR was not modulated by treatment [yoked/saline: 0.9871 ± 0.02117, nicotine: 0.9469 ± 0.02118; *t*_(59)_ = 1.263, *p* = 0.2116; Figure 3B]. In a way to assess inhibitory tone over evoked potentials the GABA_A_ receptor antagonist bicuculline (20 μM) was bath perfused. Bicuculline disinhibited PS amplitude to a similar extent in both treatment groups [yoked/saline: 138.9 ± 9.001%, nicotine: 145.8 ± 12.20%; main effect treatment: *F*_(1, 43)_ = 0.2253, *p* = 0.6374; time *F*_(15, 645)_ = 1.948, *p* = 0.0168; time × drug *F*_(15, 645)_ = 0.6246, *p* = 0.8559; Figure 3C]. However, when monitoring stimulus–response curves in bicuculline-treated slices, synaptic output was significantly enhanced in rats previously receiving nicotine [main effect treatment: *F*_(1, 42)_ = 5.747, *p* = 0.0210; time *F*_(6, 252)_ = 207.5, *p* < 0.001; time × drug *F*_(6,252)_ = 0.3426, *p* = 0.9138; Figure 3D]. PPR did not differ between the treatment groups in bicuculline-treated slices [yoked/saline: 1.083 ± 0.02135, nicotine: 1.042 ± 0.01620; *t*_(44)_ = 1.357, *p* = 0.1818; data not shown].

Whole cell recordings demonstrated no significant effect on passive membrane properties. Neither membrane resistance [yoked/saline: 161.0 ± 16.96 MΩ, nicotine: 154.9 ± 18.51 MΩ; *t*_(61)_ = 0.2402, *p* = 0.8110] nor capacitance [yoked/saline: 55.97 ± 3.011 pF, nicotine: 51.31 ± 3.638 pF; *t*_(61)_ = 0.3279, *p* = 0.7441] were significantly affected by nicotine SA followed by 1 month abstinence (Figures 3F, G). Recordings of spontaneous activity demonstrated no change in the frequency of excitatory inputs [sEPSC frequency: yoked/saline: 1.336 ± 0.1510 Hz, nicotine: 1.653 ± 0.2199 Hz; *t*_(57)_ = 1.231, *p* = 0.2233; Figure 3H]. However, sEPSCs amplitude was significantly enhanced in MSNs from nicotine-exposed rats [yoked/saline: −7.552 ± 0.2276 pA, nicotine: −8.503 ± 0.3483 pA; *t*_(57)_ = 2.403, *p* = 0.0195; Figure 3I, L], with no concomitant change in rise-or decay-time [rise-time: yoked/saline: 1.394 ± 0.0762 pA, nicotine: 1.296 ± 0.0630 pA; *t*_(57)_ = 0.7588, *p* = 0.4511; decay-time: yoked/saline: 6.200 ± 0.2275 pA, nicotine: 6.043 ± 0.3616 pA; *t*_(57)_ = 0.9447, *p* = 0.3488; Figures 3H, J, K].

### A history of nicotine self-administration produces sustained changes in accumbal MSNs excitability

The increase in sEPSC amplitude and evoked potentials indicates that a history of nicotine self-administration may act to increase excitability of accumbal MSNs that persist during prolonged abstinence. In the last set of experiments, current clamp recordings were thus performed to assess changes in excitability. Recordings from yoked animals were not significantly different compared to rats self-administrating saline [delta voltage: *F*_(1, 21)_ = 1.395, *p* = 0.2508; rheobase: *t*_(21)_ = 0.6529, *p* = 0.5209; threshold: *t*_(21)_ = 0.9214, *p* = 0.3673; AP latency: *F*_(1, 21)_ = 0.1149, *p* = 0.7380; AP frequency: *F*_(1, 21)_ = 0.006849, *p* = 0.9348] and the groups were thus pooled.

**Figure 4:**
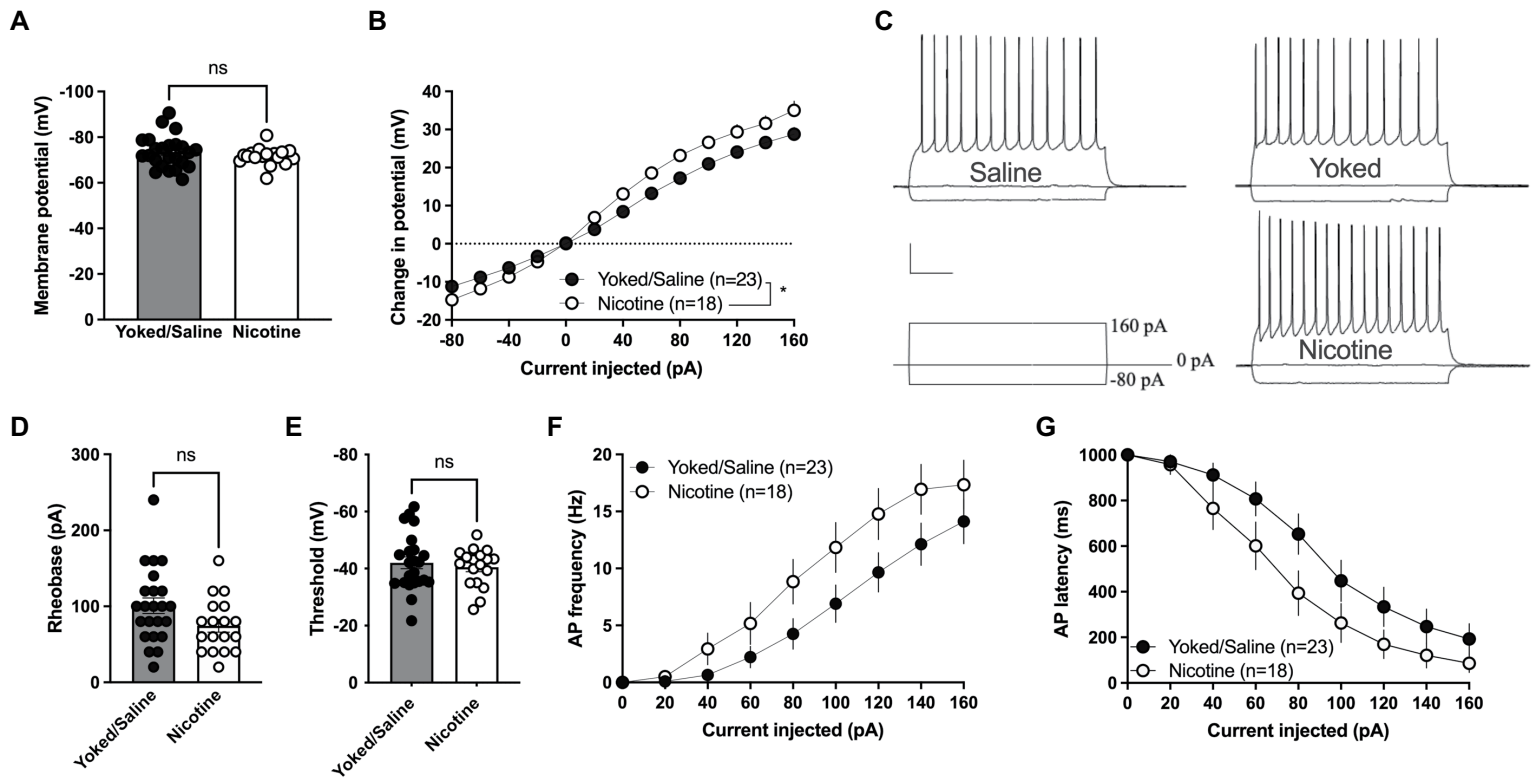
Increased excitability in nAcS MSNs after nicotine SA. **(A)** Membrane voltage was not affected by nicotine SA. **(B)** Relative change in membrane potential evoked by current injection was significantly bigger in MSNs from nicotine SA rats. **(C)** Representative traces of current clamp recordings from all treatment groups. Calibration: 0.2 s, 20 mV. **(D,E)** A trend toward reduced rheobase was observed in MSNs from nicotine SA rats, while threshold was not modulated. **(F,G)** MSNs from nicotine SA rats further demonstrated a trend toward increased AP frequency and decreased AP latency, but the effects were not significant. Values are presented as mean (±SEM), *n* = number of slices, taken from at least four animals. ^*^p < 0.05.

Membrane voltage was not significantly affected when comparing current clamp recordings performed in MSNs from yoked/saline controls with nicotine-self-administering rats [yoked/saline: −73.36 ± 1.443 mV, nicotine: −71.57 ± 0.9277 mV, student’s *t*-test: *t*_(39)_ = 0.9456, *p* = 0.3502; Figure 4A]. However, the relative change in membrane voltage (delta voltage) elicited by current injection was more pronounced in MSNs from rats self-administrating nicotine [repeated measures ANOVA, main effect treatment: *F*_(1, 39)_ = 6.097, *p* = 0.0180; time *F*_(12, 468)_ = 458.7, *p* < 0.0001; time × drug *F*_(12, 468)_ = 6.587, *p* < 0.001; Figures 4B, C]. A trend toward decreased rheobase was found in brain slices from nicotine-exposed rats [yoked/saline: 100.9 ± 10.25 pA, nicotine: 74.44 ± 8.371 pA: *t*_(39)_ = 1.920, *p* = 0.0622] but not for threshold for action potential firing [yoked/saline: −42.03 ± 2.084 mV, nicotine: −40.48 ± 1.586 mV, *t*_(39)_ = 0.5658, *p* = 0.5748; Figures 4D,E]. Action potential firing elicited by current injection showed a trend toward increased excitability [repeated measures ANOVA, main effect treatment: *F*_(1, 39)_ = 3.672, *p* = 0.0627; time *F*_(7,273)_ = 62.99, *p* < 0.0001; time × drug *F*_(7,273)_ = 1.237, *p* = 0.2825; Figure 4F], and reduced latency for AP firing [repeated measures ANOVA, main effect treatment: *F*_(1, 39)_ = 3.435, *p* = 0.0714; time *F*_(7, 273)_ = 70.88, *p* < 0.001; time × drug *F*_(7, 273)_ = 0.9778, *p* = 0.4476; Figure 4G].

## Discussion

Despite being a brain region involved in drug reinforcement, studies in the nAcS that outline neuroplasticity elicited by nicotine self-administration are scarce. In fact, this study is to our knowledge the first to monitor sustained neuroadaptations in the nAc after a period of nicotine-self-administration followed by 1 month of forced abstinence. We show that after prolonged forced abstinence from nicotine self-administration excitatory synapses in the nAcS are altered. We observed an increase in evoked field potentials and sEPSC amplitude in animals with a history of contingent nicotine SA potentially indicating higher excitability of accumbal MSNs compared to control. This finding was further supported by current clamp recordings, showing a strong trend toward reduced rheobase and AP latency, with a concomitant increase in AP frequency of MSNs.

Interestingly, saline self-administering rats presented an operant responding behavior maintained by nicotine paired visual stimuli. When the reinforcement effort was incremented from FR1 to FR3 saline-SA rats decreased the number of active responses to a stable level. However their active lever pressing was maintained significantly higher than the inactive lever responding and reinstated to baseline during cue-reinforced responding under extinction conditions. Other previous studies have confirmed the contribution of environmental cues in driving self-administration behavior, that when paired with contingent saline-SA produced even stronger responding than nicotine-SA in the absence of these stimuli (Donny et al., 2003; Garcia-Rivas et al., 2019). However, this mild reinforcing effect of visual cues in driving operant behavior did not produce any sustained neuroadaptations in the saline-SA group when compared to yoked-treated control within the accumbens shell. This finding indicates that sustained effects on accumbens shell neurotransmission is produced by the drug itself or the drug in combination with instrumental conditioning rather than the cue-conditioned reinforcer alone.

The lack of sustained neurophysiological transformations in the shell of saline self-injecting rats makes it a reliable control group when studying the involvement of this brain region in incubation of drug craving and relapse to environmental stimuli. On the other hand, short self-administration of saline paired with cue presentation has been shown to produce changes in neuronal activity by c-fos expression in the nAcS and other brain structures (Zahm et al., 2010). It is also possible that selective neuronal ensembles are recruited, and that we do not capture these transitions when monitoring subsets of cells in whole cell recordings or collective activity of hundreds cells through field potential recordings. In addition, it should be noted that other subregions within the striatum or the amygdala may be more robustly recruited during operant behavior invigorated by conditioned stimuli. In particular, dorsal striatal circuits encode consolidating new instrumental actions (Smith et al., 2021), and the nucleus accumbens core has been shown to be relevant in pavlovian drug seeking behavior elicited by psychostimulants (Corbit et al., 2001; Ito et al., 2004; Corbit and Balleine, 2011). Studies performed in other brain regions may thus be more sensitive to the choice of control group.

Both accumbens core and shell have been shown to be involved in abstinence-induced incubation of cocaine and methaphetamine craving (Conrad et al., 2008; Lee et al., 2013; Xi et al., 2013; Cruz et al., 2014; Scheyer et al., 2016) while evidences on nicotine are scarce. Previous studies have shown that 2 weeks of nicotine withdrawal presented c-fos activation in both core and shell while potentiation of excitatory synapses in the nAc core was observed at the same abstinence period (Gipson et al., 2013a; Funk et al., 2016). In our study, measures from whole cell voltage clamp and evoked field potential recordings showed that also excitatory synapses in the nAcS were altered after prolonged forced abstinence from nicotine self-administration. sEPSC amplitude was significantly enhanced in accumbal MSNs from rats self-administering nicotine. Since neither decay nor rise time were significantly affected it is possible that the change in amplitude is linked to an increase in the number of excitatory receptors on accumbal MSNs. While a cocktail of CNQX and APV blocked recorded sEPSCs, neurons are voltage clamped at-65 mv and the amplitude is thus most likely associated with activation of postsynaptic AMPA receptors. In addition, voltage clamp recordings were supported by field potential recordings that presented a potentiation of stimulus–response curves in slices from nicotine-SA rats. Evoked potentials in the nAcS were robustly blocked by CNQX, indicating that these recordings primarily reflect activation of AMPA receptors.

AMPA/kainate receptors has been suggested to be selectively involved in the relapse of nicotine seeking after a period of abstinence (Gipson et al., 2013b; Ruda-Kucerova et al., 2021). Interestingly, studies on cocaine seeking have shown that both accumbens shell and core undergo similar AMPAR plasticity after prolonged withdrawal and these neuroadaptations contribute to the expression of incubation of cocaine craving (Conrad et al., 2008; Ping et al., 2008; Mameli et al., 2009; McCutcheon et al., 2011). For instance, both increased AMPA receptors expression and changes in subunit composition is observed in the accumbens following withdrawal from cocaine or methamphetamine (Conrad et al., 2008; Wolf and Tseng, 2012; Scheyer et al., 2016; Murray et al., 2019). Electrophysiological studies support synaptic potentiation of nAc excitatory synapses where prolonged abstinence following cocaine, enhances AMPAR-dependent synaptic strength with both increases in mEPSC amplitude and AMPA/NMDA ratio (Kourrich et al., 2007; Ortinski et al., 2012). Our findings suggest a possibly common AMPAR nAcS metaplasticity during forced abstinence for both nicotine and cocaine beyond the other evidences of shared molecular effects within the accumbens of these two drugs of abuse (Di Chiara and Imperato, 1988; Pich et al., 1997; McClung et al., 2004; Ciccocioppo et al., 2021). Importantly negative affective states, generally experienced during late nicotine withdrawal, are characterized by increased AMPA receptor expression in nAcS selectively in dopamine D1 MSNs (Pignatelli et al., 2021) In addition, cocaine abstinence has been shown to selectively increase the rectification of AMPA receptor-mediated currents in nAcS dopamine D1 receptor expressing MSNs (Inbar et al., 2022). The data presented in this study did not distinguish between dopamine D1 and D2 receptor expressing MSNs. If withdrawal induced stress responses contribute to the transformations retrieved here, it is thus possible that more pronounced effects would have been observed if separating recordings associated with the direct or indirect pathway.

While we observed changes in sEPSC amplitude, voltage clamp recordings revealed no significant increase in the frequency of synaptic inputs onto MSNs. However, nAc shell is innervated by glutamatergic projections arising from several distinct areas including the infralimbic cortex, basal amygdaloid complex, hippocampus and ventral prelimbic cortex (Voorn et al., 2004). It is thus possible that changes in specific inputs exist, but that these transformations are shielded by inputs arising from other regions.

Changes in neuronal excitability of nAc MSNs are critical for the manifestation of behavioral alterations related to drugs of abuse (Hu et al., 2004; Dong et al., 2006; Mu et al., 2010; Liu et al., 2012; Ma et al., 2013), and the data presented here indicated an increased excitability of MSNs in nicotine-SA male rats. Injected current produced a significantly greater change in membrane voltage, and there was a strong trend toward increased AP frequency and reduced rheobase and AP latency. This is partially supported by a previous study showing that nicotine addicted mice presented hyperexcitability of nAcS MSNs due to a decrease in potassium conductance mediated by large-conductance Ca^2+^-activated K+ channels (Ma et al., 2013). Our observation of increased excitability of nAcS during nicotine forced abstinence in male rats might further be a factor that could drive relapse to nicotine seeking. One limitation of the study is that only male rats were used. Considering that women experience more severe withdrawal symptoms including greater negative affect compared to men (Piasecki et al., 2003; Pang and Leventhal, 2013; O'Dell and Torres, 2014), nicotine abstinence-induced neuroadaptations may be more pronounced in female rats. Future research in both sexes will be essential to prove a functional role of the presented shell MSNs alterations and nicotine seeking behavior.

In conclusion, our findings suggest that a history of nicotine self-administration produces sustained neuroadaptations in the nAcS while operant responding driven by nicotine visual stimuli has no long-term effects on MSNs in the nAcS. The observed neuronal alterations in excitatory activity of nAcS MSNs during nicotine forced abstinence suggest that normalization of these synapses activity has implications for the treatment of nicotine seeking and relapse prevention.

## Data availability statement

The raw data supporting the conclusions of this article will be made available by the authors, without undue reservation.

## Ethics statement

Procedures were conducted in accordance with the National Committee for Animal Research in Sweden and approved by the Local Ethics Committee for Animal Care and Use at Gothenburg University: ID 2510, decision 25/09/2019.

## Author contributions

AD and LA were responsible for the study concept and design. AD designed and performed the behavioral and field potential electrophysiological experiments, collected and analyzed the data, and wrote and revised the manuscript. LA contributed in analyzing, interpreting, and assembling the data and revising the manuscript. EL and DC performed and analyzed whole-cell electrophysiological recordings. All authors contributed to the article and approved the submitted version.

## Funding

This work was supported by the Swedish Research Council (vetenskapsrådet: 2018-02814 and 2020-00559), Governmental support under the ALF agreement (ALFGBG-966287), and Fredrik and Ingrid Thurings Stiftelse (2020-00593).

## Conflict of interest

The authors declare that the research was conducted in the absence of any commercial or financial relationships that could be construed as a potential conflict of interest.

## Publisher’s note

All claims expressed in this article are solely those of the authors and do not necessarily represent those of their affiliated organizations, or those of the publisher, the editors and the reviewers. Any product that may be evaluated in this article, or claim that may be made by its manufacturer, is not guaranteed or endorsed by the publisher.
